# Structure Optimization and Data Processing Method of Electronic Nose Bionic Chamber for Detecting Ammonia Emissions from Livestock Excrement Fermentation

**DOI:** 10.3390/s24051628

**Published:** 2024-03-01

**Authors:** Yeping Shi, Yunbo Shi, Haodong Niu, Jinzhou Liu, Pengjiao Sun

**Affiliations:** 1 The Higher Educational Key Laboratory for Measuring & Control Technology and Instrumentation of Heilongjiang Province, Harbin University of Science and Technology, Harbin 150080, China; 1810600004@stu.hrbust.edu.cn (Y.S.); 2010600001@stu.hrbust.edu.cn (H.N.); liujinzhou0709@outlook.com (J.L.); 2 Electronics and Communication Engineering School, Jilin Technology College of Electronic Information, Jilin 132021, China; 1710600004@stu.hrbust.edu.cn; 3 Heilongjiang Province Key Laboratory of Laser Spectroscopy Technology and Application, Harbin University of Science and Technology, Harbin 150080, China; 4 National Experimental Teaching Demonstration Center for Measurement and Control Technology and Instrumentation, Harbin University of Science and Technology, Harbin 150080, China

**Keywords:** active pumping, electronic nose system, biomimetic chamber, ammonia detection, backpropagation (BP) neural network algorithm

## Abstract

In areas where livestock are bred, there is a demand for accurate, real-time, and stable monitoring of ammonia concentration in the breeding environment. However, existing electronic nose systems have slow response times and limited detection accuracy. In this study, we introduce a novel solution: the bionic chamber construction of the electronic nose is optimized, and the sensor response data in the chamber are analyzed using an intelligent algorithm. We analyze the structure of the biomimetic chamber and the surface airflow of the sensor array to determine the sensing units of the system. The system employs an electronic nose to detect ammonia and ethanol gases in a circulating airflow within a closed box. The captured signals are processed, followed by the application of classification and regression models for data prediction. Our results suggest that the system, leveraging the biomimetic chamber, offers rapid gas detection response times. A high classification prediction accuracy, with a determination coefficient R^2^ value of 0.99 for single-output regression and over 0.98 for multi-output regression predictions, is achieved by incorporating a backpropagation (BP) neural network algorithm. These outcomes demonstrate the effectiveness of the electronic nose, based on an optimized bionic chamber combined with a BP neural network algorithm, in accurately detecting ammonia emitted during livestock excreta fermentation, satisfying the ammonia detection requirements of breeding farms.

## 1. Introduction

Ammonia (NH_3_) is a major pollutant in densely populated livestock breeding areas and is recognized by the United States Environmental Protection Agency (EPA) as one of 366 extremely hazardous substances. NH_3_ is a potent-smelling gas [1]. It is released into the environment through the fermentation of livestock excreta, including urine and feces [2]. High ammonia levels in breeding farms pose potential health threats to both humans and livestock [3]. It readily adsorbs onto the skin, mucous membranes, and eye conjunctiva, causing irritation and inflammation. In humans, ammonia exposure can cause various health problems, including ocular, respiratory, pulmonary, and skin diseases [4,5,6]. Prolonged exposure to elevated ammonia levels can adversely impact livestock growth, productivity [7], fertility, and egg production [8]. Recce et al. [9] demonstrated that broiler chickens exposed to ammonia volumes of 25–50 ppm experience slower weight gain and increased mortality rates, further escalating at 100–200 ppm. The recommended upper limit for ammonia gas volume is 25 ppm [10]. Therefore, maintaining accurate, real-time, and stable monitoring of ammonia concentrations during breeding is essential. This approach facilitates the setting of alarms to protect humans from prolonged exposure to ammonia, enhance breeding efficiency, ensure compliance with environmental regulations, and support sustainable agricultural practices.

Various ammonia detection methods exist, including calorimetry [11,12,13], electrochemistry [14,15,16], ion-selective electrodes [17,18,19,20], gas-sensitive tubes [21,22,23], spectroscopy [24,25,26], and sampling with laboratory analysis [27,28,29]. Among them, artificial olfaction systems, or electronic noses, have emerged as a mainstream technology in recent years, particularly for ammonia detection in breeding farms. These systems offer real-time, non-invasive, and automated ammonia concentration measurements, eliminating the need for manual sampling or laboratory analysis [30]. Electronic noses boast many advantages, such as high sensitivity, instantaneous monitoring, portability, ease of use, and cost-effectiveness, as they negate the need for expensive reagents, specialized equipment, or trained personnel. Recent research highlights the potential of electronic noses in detecting ammonia in livestock breeding environments.

The development of electronic nose technology has revolutionized ammonia detection in livestock breeding environments. A pivotal study [31] designed an intelligent electronic nose system to measure and analyze odors in these settings. They found a significant correlation coefficient (R) of 0.932 between the odor concentrations obtained using artificial neural networks and the actual concentrations. In [32], an electronic nose was employed for the rapid online detection of the volatile organic compound (VOC) concentration of garbage. The results from gas chromatography-mass spectrometry and the electronic nose were strongly related, as evidenced by t-tests and F-tests. In another study [33], an electronic nose based on a CNT-TiO_2_ sensor was proposed to measure VOCs gas pollution indoors. Four different VOCs gases were classified using support vector machine (SVM) algorithms, and the accuracy rate was 97.5%. In a different approach, researchers developed a livestock odor monitoring system incorporating information and communications technology (ICT) and ammonia sensors [34], enabling trend analysis of collected odor data. Furthermore, a study applied electronic nose technology [35] in pigsties by integrating a deep learning algorithm optimized using particle swarm optimization to enhance detection accuracy, achieving a determination coefficient R^2^ over 0.94. However, most electronic nose methods employ passive sensor placement, resulting in poor real-time data and detection accuracy.

We present a novel approach, which is an active pumping electronic nose system, incorporating a biomimetic gas collection chamber for more effective ammonia gas detection. Computational fluid dynamics (CFD) was adopted to analyze airflow over sensor surfaces in various chamber and printed circuit board (PCB) structures. We conducted a comparative analysis using data from eight sensors of the electronic nose. For classification and prediction of ammonia and ethanol detection, we utilized three models: a backpropagation (BP) neural network, a long short-term memory (LSTM) network, and a particle swarm optimized BP neural network (PSO-BP). In addition, we applied three multiple-output regression analysis models—a BP neural network, a deep neural network (DNN), and a least squares support vector machine (LSSVM )—to predict volume concentrations of ammonia and ethanol. The chosen model is validated using the new dataset. Our results confirm that the electronic nose, enhanced via the biomimetic sensor system, significantly improves detection performance, contributing to precise, real-time, and stable monitoring of ammonia concentrations in livestock breeding environments.

## 2. Design of Electronic Nose System

### 2.1. Structural Design of Electronic Nose System

**Figure 1:**
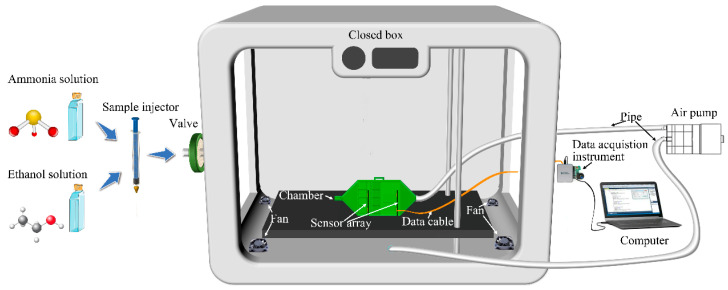
Schematic of the electronic nose detection experimental system.

The experimental setup for the electronic nose detection system is illustrated in Figure 1, featuring a comprehensive assembly that includes an ammonia solution, an ethanol solution, a sample injector needle, a closed box, a sensor array, a biomimetic chamber, an air pump, fans, a data acquisition device, and a computer. The design ensures a constant gas volume concentration within the box and sufficient contact between the test gas and the sensor. This is achieved by pumping the airflow using an air pump, with the sensor array placed inside the biomimetic chamber. The intake end of the air pump was connected to the outlet of the biomimetic chamber via a pipe. The inlet of the biomimetic chamber is the starting point for the circulating airflow, creating a circulating airflow between the inlet and the outlet of the chamber. The gas concentration within this circulating airflow mirrors that inside the closed box.

### 2.2. Experimental Materials and Equipment

**Figure 2:**
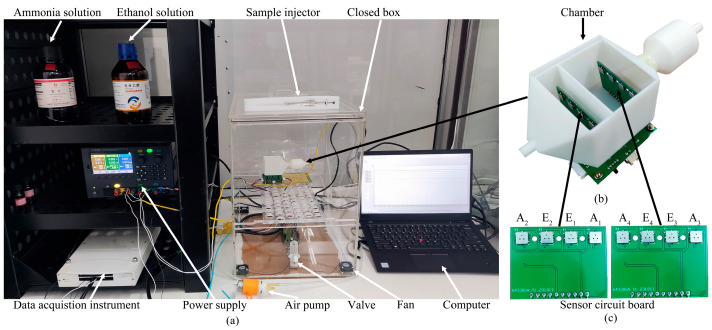
Physical setup of the electronic nose detection experimental system. (**a**) Detection experiment system; (**b**) Bionic chamber; (**c**) Sensor arrays.

Figure 2 presents the physical setup of the experimental system. In Figure 2a, we observe the use of analytical-grade ammonia solution with a 25% NH_3_ content and anhydrous ethanol solution with a mass fraction of ≥99.7%. The ranges of the micro-injectors are 10 and 25 μL, respectively. The volume of the closed box is 48 L (0.3 × 0.4 × 0.4 m). The test gas volume concentration inside the box is controlled by injecting measured amounts of ammonia and ethanol solutions. The data acquisition instrument shown in Figure 2a is a National Instruments USB6289 (Emerson Electric Co., St Louis, USA) with a 10 Hz sampling frequency. A KEYSIGHT E36313A power supply (Keysight Technologies Inc., Santa Rosa, CA, USA) provides a constant 5 V voltage to the system. The air pump shown in Figure 2a is a micro diaphragm pump model EDLP600-D12B (Kamoer Fluid Tech (Shanghai) Co., Ltd., Shanghai, China ) and it controls the flow rate by adjusting the input voltage, with the rate measured at 650 SCCM using a glass rotor flowmeter model LZB-4WB (Xinghua xiangjin flow meter factory, Taizhou, China).

In Figure 2b, the biomimetic chamber, made from white photosensitive resin with a 0.1 mm accuracy, is created using an SLA880 device from ZRapid Tech (Suzhou, China). The chamber includes a flow guide cavity to ensure stable airflow, featuring a 30 mm inner diameter in the body portion and a 3 mm inner diameter at both the inlet and the outlet. Figure 2c shows two sets of sensor arrays inside the biomimetic chamber. Each set contains four sensors, symmetrically arranged along the central axis of the chamber. A_1–4_ sensors are designated for ammonia gas, while E_1–4_ are for ethanol detection. The models for these sensors are SMD1002 for ammonia and SMD1005 for ethanol. The ammonia and ethanol sensors are both sourced from Suzhou Huiwen Nano S&T Co., Ltd. (Suzhou, China). The ammonia sensor can detect gas volume concentrations ranging from 0 ppm to 300 ppm with a resolution of 1 ppm, while the ethanol sensor can detect concentrations from 0 ppm to 500 ppm with a finer resolution of 0.2 ppm. The sensors were subjected to a preheating period of 48 h before use. Each experimental run involves a 3 min data collection phase followed by a 10 min purge of the system with dry air to prepare for the subsequent experiment.

### 2.3. Design of Biomimetic Chamber

**Figure 3:**
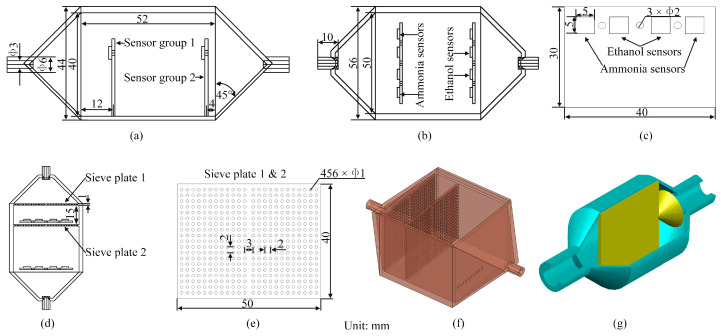
Model of the electronic nose biomimetic chamber and sensor array: (**a**) Left view of the chamber; (**b**) Top view of the chamber; (**c**) Front view of the sensor array; (**d**) Top view of the chamber with sieve plate; (**e**) Front view of the sieve plate; (**f**) Chamber model; (**g**) Flow guide cavity model.

The design of the electronic nose’s biomimetic chamber was inspired by the olfactory functions and sensitivity of mammals, particularly the unique airflow patterns in their olfactory processes. These patterns are determined by the olfactory recesses located in the posterior part of the nasal cavity [36]. Based on the structure of canine olfactory-sensitive nasal cavities [37,38] and relevant studies [39], the chamber was designed to emulate a “large chamber with a small entrance,” including a sieve plate structure. This design mirrors the distribution of olfactory cells in the nasal cavity. The biomimetic chamber houses a sensor array at both its front and rear ends, akin to the placement of olfactory cells in the nasal cavity. In front of each sensor array, a sieve plate mimics the turbinate bone structure found in canine noses. Moreover, 2 mm diameter vent holes were designed between adjacent sensors on the PCB where the sensors were placed to increase gas flow near the sensors. A flow guide cavity at the rear end of the biomimetic chamber ensures consistent airflow within. Figure 3 illustrates various aspects of the biomimetic chamber and PCB design. Figure 3a shows the left view of the electronic nose chamber, with the chamber’s dimensions of 40 × 52 × 50 mm and PCB placement. Both the inlet and outlet of the chamber are 3 mm in diameter and are extended to 10 mm long hollow cylinders. Figure 3b presents a top view of the chamber, indicating the lateral dimensions and sensor positions, with sensors in both groups sharing the same layout. Figure 3c shows the dimensions of the PCB (40 × 30 × 1.2 mm) and sensors (5 × 5 × 1 mm). Figure 3d shows the position of the sieve plate inside the chamber, with a 15 mm gap between the two plates. Figure 3e displays the design of the sieve plates, each 1 mm thick with 1 mm diameter holes spaced 2 mm apart center-to-center. Figure 3f represents a three-dimensional (3D) view of the chamber model, while Figure 3g illustrates the internal 3D view of the flow guide cavity at the chamber’s rear. The dimensions of the inlet and outlet of this cavity match those of the biomimetic nasal cavity, featuring a 30 mm inner diameter. The design includes two chamber variations: “Chamber A” without the sieve plate, as shown in Figure 3a, and “Chamber B” with the sieve plate, as shown in Figure 3d.

## 3. Results and Discussion

### 3.1. Simulation Results of Detection Chambers

ANSYS (2021 R1) analysis was utilized to examine the flow field near the sensor surfaces within the detection chambers, focusing on the airflow pressures experienced by sensors in Chambers A and B. Additionally, the study compared the airflow velocities near the sensors in Chamber B using both perforated and non-perforated PCBs. The boundary condition for inlet flow velocity was set at −1.53 m/s, which corresponds to an active pumping rate of 650 SCCM at the outlet of the biomimetic chamber.

**Figure 4:**
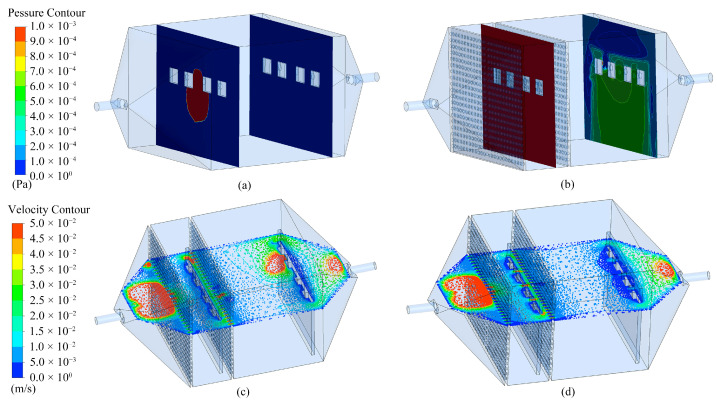
Comparison of simulation results: (**a**) Pressure contour of sensors in Chamber A; (**b**) Pressure contour of sensors in Chamber B; (**c**) Gas velocity contour in Chamber B with perforated PCB; (**d**) Gas velocity contour in Chamber B with non-perforated PCB.

Figure 4a,b depicts pressure contours along the longitudinal sections of the two chambers. In Chamber A, as illustrated in Figure 4a, the absence of a sieve plate enables substantial airflow to rapidly reach the sensor surfaces located along the central axis at the chamber inlet, resulting in uneven pressure distribution on the sensor array. Sensors at the chamber inlet experience high pressure. In contrast, Figure 4b shows that in Chamber B the sieve plate causes the airflow to disperse into multiple smaller streams through the holes before reaching the sensor surfaces, resulting in a more uniform pressure distribution. Sensors at the outlet of Chamber B are subjected to higher pressure compared to those in Chamber A, enhancing contact and interaction with gas molecules and, thus, increasing the response value. Figure 4c,d shows the velocity contours across the transverse section of Chamber B. Figure 4c shows that the sensor surface at the chamber inlet experiences lower airflow velocity owing to obstructions by the PCB, causing airflow to redirect toward the gaps on the sides and top of the PCB. The airflow velocity is non-uniform at the sensor array at the chamber outlet, with significant variations. As shown in Figure 4d, some airflow passes through the holes on both sides of the sensor, flowing toward the rear of the chamber, facilitating interactions between the sensor and the test gas. The velocity distribution on the sensor array is nearly uniform, minimizing the impact of airflow velocity on the sensor response. Consequently, the biomimetic chamber with sieve plates and the perforated PCB design ensure a uniform gas flow field, enhancing sensor response.

### 3.2. Gas-Sensitive Response of Electronic Nose System

**Figure 5:**
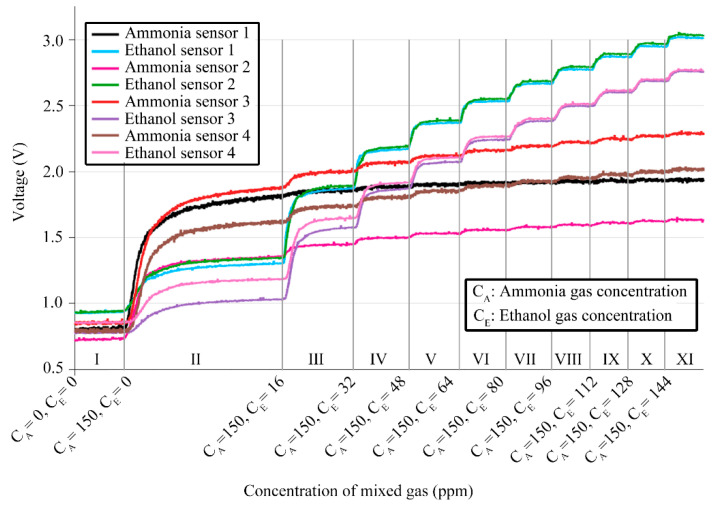
Real-time response signal of sensor array.

Figure 5 illustrates the response curves of the eight gas sensors in the electronic nose system, corresponding to different volume concentrations of ammonia and ethanol gases within the closed box. The x-axis represents the volume concentrations of ammonia and ethanol gases, converted from liquid concentrations, measured in ppm. The y-axis represents the response of the electronic nose system in volts (V). The output voltage of the system is given by

(1) $$U_{out}=\frac{Vcc}{R_{S}+R_{L}}\cdot R_{L},$$

where $U_{out}$ indicates the output voltage of the electronic nose system, Vcc = 5 V, $R_{S}$ indicates the resistance of the sensor, and $R_{L}$ indicates the adjustable resistance connected in series with the sensor. In this experimental system, adjusting $R_{L}$ ensures that the output voltage of the electronic nose system in air is between 0.88 V and 1.1 V.

The experiment was divided into several phases. In Phase I, both ammonia and ethanol gas concentrations within the closed box were 0 ppm. During Phase II, the ammonia solution was introduced, resulting in a concentration of 150 ppm for ammonia, while ethanol remained at 0 ppm, over a period of 5 min. From Phases III to XI, ethanol liquid, corresponding to a 16 ppm gas volume concentration, was incrementally injected into the chamber, maintaining a constant ammonia gas concentration, over 3 min intervals. The sensor response time was observed to be less than 30 s, with each sensor producing a distinct response magnitude in response to both gases. Following three repeated measurements, the response curves of the electronic nose was found to be at the same level.

The electronic nose system contains metal oxide semiconductor sensors, which typically suffer from poor selectivity, meaning a single gas can trigger responses from multiple sensors. The results from Figure 5 indicate that both ammonia and ethanol gas sensors reacted specifically to each other’s gases. The sensitivity of the ammonia sensor to ethanol gas was minimal when the concentration of the target gas reached a certain level.

The response of an electronic nose typically increases with higher volume concentrations of ammonia and ethanol gases, yet a direct correlation between the two is not always evident. As illustrated in Figure 5, the response amplitudes for ammonia and ethanol sensors vary, even at identical gas concentrations. This variation can be attributed to several factors.

- First, the series resistance R_L_ of the sensor given by Equation (1) has a significant impact on the output voltage of the electronic nose.
- Second, the sensitivity characteristics of the sensors play a crucial role. These characteristics are not solely determined by the inherent properties of the sensor but also by external environmental factors, such as temperature and humidity, as well as the aging time of the sensor. Figure 3d illustrates how aging affects the sensor’s baseline resistance R_0_ and the resistance R_S_ in the presence of ammonia, resulting in minor changes that can alter the response of the sensor.
- Additionally, the effect of airflow on the sensor is a critical factor [39,40]. Previous studies have shown that a sensor’s response is linked to the flow velocity of dynamic airflow. Schuyler et al. [41,42] proposed an algorithm to correct the impact of dynamic airflow velocity on sensors during ammonia detection.

Given these complexities, it is essential to implement artificial intelligence techniques for the classification and prediction of ammonia and ethanol gases in a regressive manner based on the data collected by the active pumping electronic nose system.

### 3.3. Intelligent Recognition of Electronic Nose Response

In this study, the data collected by the electronic nose system were first preprocessed. Subsequently, a series of models were employed for data analysis: three classification prediction models, three single-output regression prediction models, and three multi-output regression prediction models.

#### 3.3.1. Data Preprocessing

The preprocessing of the output signals from the electronic nose involves a two-step process.

- The first step involves dividing the response voltage of the electronic nose, when measuring the target gas, by the response voltage of the electronic nose in air, which is expressed in Equation (2):

(2) $$n_{i}=\frac{{\bar{U}}_{\mathrm{gas},i}}{{\bar{U}}_{0,i}},$$

where $n_{i}$ indicates the processed result for the response voltage of the *i*-th sensor, ${\bar{U}}_{\mathrm{gas},i}$ denotes the average output voltage of the *i*-th sensor after detecting the target gas for 10 s, and ${\bar{U}}_{0,i}$ indicates the output voltage of the *i*-th sensor in air.

- The second step in preprocessing is the normalization of the dataset. This is achieved through a normalization algorithm described in Equation (3) to form the test set samples:

(3) $$n^{\prime }i=(n_{i}-\min(n_{i}))/(\max(n_{i})-\min(n_{i})),$$

where $n^{\prime }i$ is the normalized results of the *i*-th sensor and $n_{i}$ indicates the data of the *i*-th sensor processed using Equation (2).

**Figure 6:**
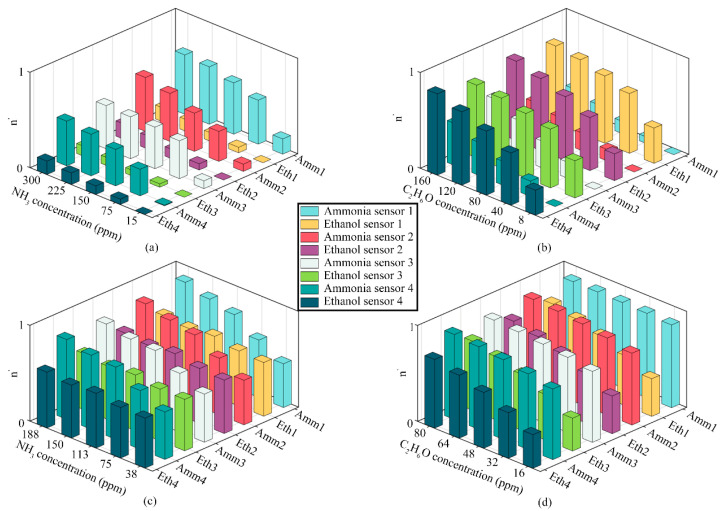
Bar graphs of normalized system output data: (**a**) C_A_ = 15, 75, 150, 225, and 300 ppm; C_E_ = 0 ppm. (**b**) C_A_ = 0 ppm; C_E_ = 8, 40, 80, 120, and 160 ppm. (**c**) C_A_ = 38, 75, 113, 150, and 188 ppm; C_E_ = 48 ppm. (**d**) C_A_ = 188 ppm; C_E_ = 16, 32, 48, 64, and 80 ppm. C_A_: Ammonia volume concentration. C_E_: Ethanol volume concentration.

Figure 6 illustrates the outcome of the preprocessing in bar graphs that represent normalized output data of the electronic nose system. The x-axis of the graph lists the sensors, which include four ammonia sensors and four ethanol sensors. The y-axis represents the volume concentrations of ammonia and ethanol gases in the closed box, converted from liquid concentrations and measured in ppm. The z-axis represents the normalized response data from the eight sensors in the electronic nose system. Figure 6 presents various scenarios. Figure 6a shows the response at different ammonia gas concentrations (15, 75, 150, 225, and 300 ppm) without ethanol. Figure 6b depicts the response for varying ethanol gas concentrations (8, 40, 80, 120, and 160 ppm) without ammonia. In Figure 6c, both gases are present, with ammonia concentrations ranging from 38 to 188 ppm and ethanol at a constant 48 ppm. Figure 6d displays the response with ethanol concentrations between 16 ppm and 80 ppm and a constant ammonia concentration of 188 ppm.

Preprocessing the original response signals from the sensor array is a critical step in enhancing the accuracy of target gas identification in electronic nose systems. This process helps mitigate interference from signals with varying response levels during network training. Equation (2) plays a crucial role in reducing the impact of time-variant characteristics of sensor sensitivity on the system response. Furthermore, the normalization using Equation (3) ensures that the input data in the prediction network are adjusted to a uniform scale. This standardization significantly improves the performance and accuracy of the model, enabling it to capture data characteristics and reduce the rate of detecting data errors more effectively.

#### 3.3.2. Analysis of Gas Classification and Prediction Results

In this study, three distinct classification algorithms were employed to train the same dataset: backpropagation (BP), long short-term memory (LSTM), and particle swarm-optimized backpropagation (PSO-BP). Preprocessed data from eight groups were input into each model. The output classification was divided into three categories: A for ammonia, B for ethanol, and C for a combination of ammonia and ethanol. All algorithms underwent training on the same dataset and were tested for their classification accuracy using identical data.

For the gas classification analysis, a raw dataset comprising 117 groups of test data was used. The dataset was randomly split into 82 training sets and 35 testing sets using random numerical values. The training sets were used to construct the model structure, initialize model parameters, and train the model. After training, the performance of the model was validated using the test set, with results collected for statistical analysis.

**Figure 7:**
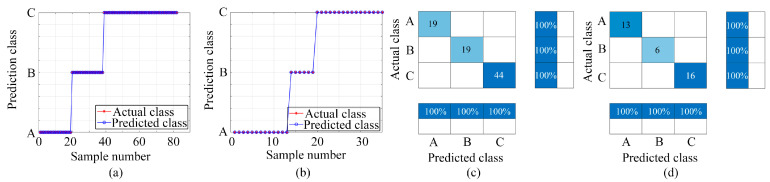
Classification and prediction results based on BP neural network (Class A: ammonia, Class B: ethanol, and Class C: ammonia and ethanol): (**a**) Training set prediction results; (**b**) Test set prediction results; (**c**) Training set confusion matrix; (**d**) Testing set confusion matrix.

**Figure 8:**
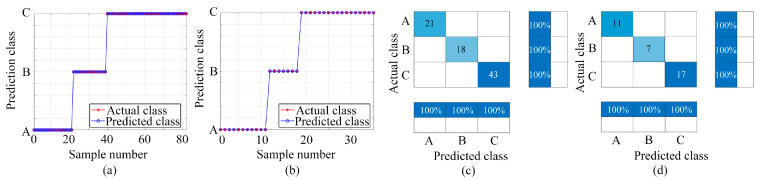
Classification and prediction results based on LSTM (Class A: ammonia, Class B: ethanol, and Class C: ammonia and ethanol): (**a**) Training set prediction results; (**b**) Test set prediction results; (**c**) Training set confusion matrix; (**d**) Testing set confusion matrix.

**Figure 9:**
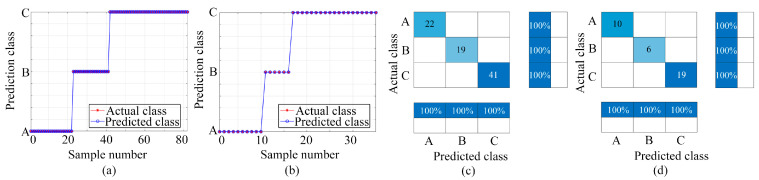
Classification and prediction results based on PSO-BP (Class A: ammonia, Class B: ethanol, and Class C: ammonia and ethanol): (**a**) Training set prediction results; (**b**) Test set prediction results; (**c**) Training set confusion matrix; (**d**) Testing set confusion matrix.

Figure 7, Figure 8 and Figure 9 display the outcomes achieved using the three classification models. Figure 7, Figure 8 and Figure 9 present the classification results using the BP, LSTM, and PSO-BP models, respectively. Each figure includes subplots: (a) Showcasing predicted results for the training set; (b) Displaying predictions for the test set; (c) Exhibiting the confusion matrix for the training set; (d) Presenting the confusion matrix for the test set—Class A: ammonia, Class B: ethanol, and Class C: ammonia and ethanol.

The effectiveness of machine learning algorithms in classification tasks is gauged via their classification accuracy, which is a metric that reflects the proportion of correctly predicted samples in relation to the total sample count. The predicted classification counts for ammonia, ethanol, and mixed gas in the dataset differ owing to the random assignment of samples in the training set and the test set. As shown in Figure 7, Figure 8 and Figure 9, the sample numbers for ammonia, ethanol, and mixed gas are 32, 25, and 60, respectively. In the BP classification method, the number of samples of A, B, and C in the training set is 19, 19, and 44, respectively, whereas the number of samples of A, B, and C in the test set is 13, 16, and 16, respectively. In the LSTM classification method, the sample numbers of A, B, and C in the training set are 21, 18, and 43, respectively, while in the test set they are 11, 7, and 17, respectively. In the PSO-BP classification method, the sample numbers for A, B, and C in the training set are 22, 19, and 41, respectively, while the sample numbers for A, B, and C in the test set are 10, 6, and 19, respectively.

In this study, the classification analysis using BP, LSTM, and PSO-BP models demonstrated remarkable effectiveness, achieving 100% accuracy in both the training and test sets, as shown in Figure 7, Figure 8 and Figure 9. These findings underscore the proficiency of the BP, LSTM, and PSO-BP models in categorizing the samples within the dataset, suggesting their reliable capability in making predictions for unknown data.

#### 3.3.3. Analysis of Single-Output Regression Prediction Results for Gas Volume Concentration

In addition to classification, the study also deployed three regression prediction models—BP, LSTM, and PSO-BP—for single-output regression prediction of gas volume concentration. These models are consistent with those used for gas classification and prediction, highlighting the versatility of these algorithms. The preprocessed data from the electronic nose served as the input for these models, with the outputs being the volume concentrations of ammonia and ethanol gases. All three algorithms underwent training and testing on the same dataset, ensuring consistency in evaluation.

**Figure 10:**
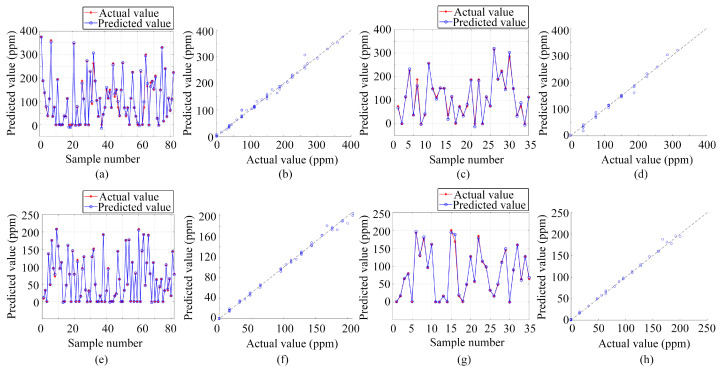
Regression prediction results based on the BP neural network: (**a**) Ammonia training set prediction results; (**b**) Scatter plot of predicted and actual values for the ammonia training set; (**c**) Ammonia test set prediction results; (**d**) Scatter plot of predicted and actual values for the ammonia test set; (**e**) Ethanol training set prediction results; (**f**) Scatter plot of predicted and actual values for the ethanol training set; (**g**) Ethanol test set prediction results; (**h**) Scatter plot of predicted and actual values for the ethanol test set.

**Figure 11:**
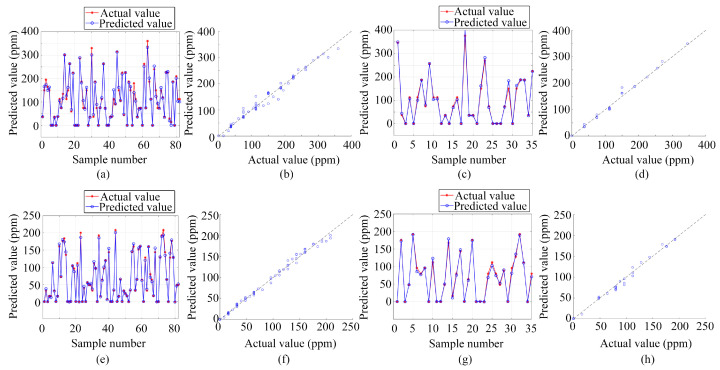
Regression prediction results based on LSTM: (**a**) Ammonia training set prediction results; (**b**) Scatter plot of predicted and actual values for the ammonia training set; (**c**) Ammonia test set prediction results; (**d**) Scatter plot of predicted and actual values for the ammonia test set; (**e**) Ethanol training set prediction results; (**f**) Scatter plot of predicted and actual values for the ethanol training set; (**g**) Ethanol test set prediction results; (**h**) Scatter plot of predicted and actual values for the ethanol test set.

**Figure 12:**
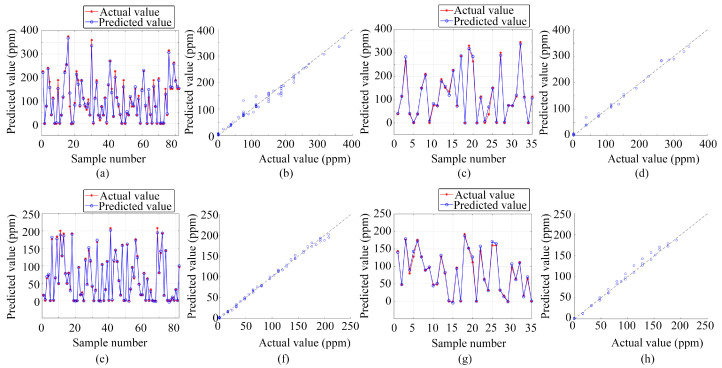
Regression prediction results based on PSO-BP: (**a**) Ammonia training set prediction results; (**b**) Scatter plot of predicted and actual values for the ammonia training set; (**c**) Ammonia test set prediction results; (**d**) Scatter plot of predicted and actual values for the ammonia test set; (**e**) Ethanol training set prediction results; (**f**) Scatter plot of predicted and actual values for the ethanol training set; (**g**) Ethanol test set prediction results; (**h**) Scatter plot of predicted and actual values for the ethanol test set.

Figure 10, Figure 11 and Figure 12 present the results obtained from these regression prediction models. Figure 10, Figure 11 and Figure 12 present the results from the BP, LSTM, and PSO-BP models, respectively. Each figure contains several subplots: (a) Predictions for the ammonia training set; (b) Scatter plots comparing predicted and actual values for the ammonia training set; (c) Predictions for the ammonia test set; (d) Scatter plots for the ammonia test set; (e) Predictions for the ethanol training set; (f) Scatter plots for the ethanol training set; (g) Predictions for the ethanol test set; (h) Scatter plots for the ethanol test set. As depicted in Figure 10, Figure 11 and Figure 12, most of the predicted values were close to the actual values in the training and testing sets when utilizing regression analysis methods, such as BP, LSTM, and PSO-BP. The scatter plots indicate that the predictive performance of the BP model surpasses that of the LSTM and PSO-BP models.

Table 1 presents the evaluation metrics for the single-output estimation model of gas volume concentration, including the determination coefficient (R^2^), mean absolute error (MAE), and root mean square error (RMSE). The R^2^ value, a key statistical measure, indicates the extent to which the variance in the observed data is predictable from the model. With R^2^ values exceeding 0.98 for all three regression prediction models, this denotes a strong fit between the data collected using the active pumping electronic nose system and the applied regression models, suggesting a high degree of consistency between the predicted and the observed values. MAE and RMSE are crucial in measuring the deviation between model predictions and actual observations, with lower values indicating higher accuracy. Considering this, the BP model outperformed the others, registering the smallest errors in regression prediction based on the electronic nose dataset. Therefore, for single-output regression prediction of gas volume concentration, the BP model not only exhibits the best fit but also the smallest error margins among the three models.

#### 3.3.4. Analysis of Multi-Output Regression Prediction Results for Gas Volume Concentration

**Figure 13:**
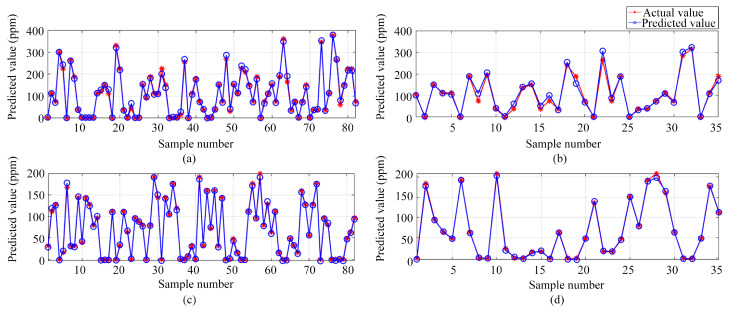
Multi-output regression prediction based on the BP neural network: (**a**) Ammonia training set predictions; (**b**) Ammonia test set predictions; (**c**) Ethanol training set predictions; (**d**) Ethanol test set predictions.

**Figure 14:**
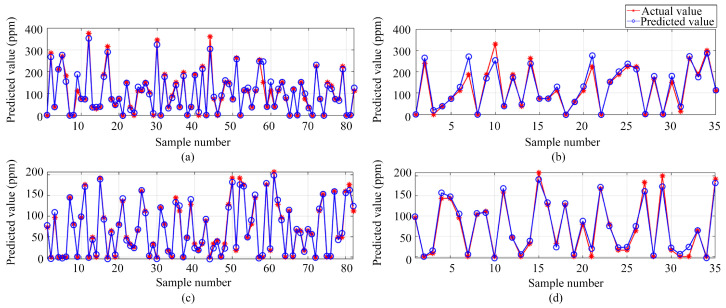
Multi-output regression prediction based on DNN: (**a**) Ammonia training set predictions; (**b**) Ammonia test set predictions; (**c**) Ethanol training set predictions, (**d**) Ethanol test set predictions.

**Figure 15:**
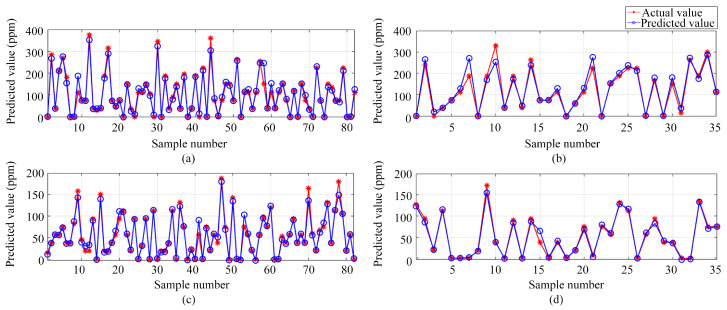
Multi-output regression prediction based on LSSVM: (**a**) Ammonia training set predictions; (**b**) Ammonia test set predictions; (**c**) Ethanol training set predictions; (**d**) Ethanol test set predictions.

Transitioning from single-output to multi-output regression, three different models were employed (i.e., BP, DNN, and LSSVM). These models aimed to predict the volume concentrations of both ammonia and ethanol gases, using the preprocessed data from the electronic nose as inputs. Figure 13, Figure 14 and Figure 15 illustrate the predictive results from these models. Figure 13, Figure 14 and Figure 15 show the results of the BP, DNN, and LSSVM models, respectively. The subplots within these figures encompass predictions for both ammonia and ethanol in training and test sets.

Table 2 presents the evaluation metrics for the multi-output estimation models and reveals that the BP model emerged with the highest accuracy, as indicated by the R^2^ value closest to 1 and the lowest MAE and RMSE. This underscores the effectiveness of the BP model in accurately predicting gas volume concentrations in a multi-output regression scenario. However, the MAE and RMSE values for the BP multi-output model are higher than those of its single-output counterpart.

#### 3.3.5. Analysis of Repeatability Test Based on BP Model

**Figure 16:**
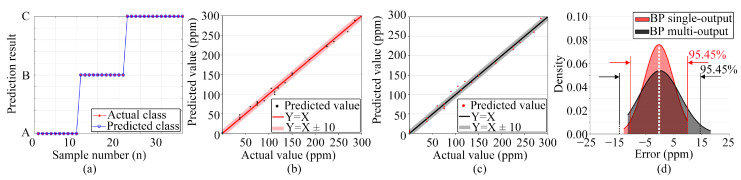
Prediction results based on the BP model: (**a**) Representing the categorization prediction results, where: Class A: ammonia, Class B: ethanol, and Class C: ammonia and ethanol; (**b**) Ammonia predictive value scatter plot based on BP model single output; (**c**) Ammonia predictive value scatter plot based on BP model multi-output; (**d**) Absolute error probability density normal distribution based on BP model predictive value.

We collected 35 new datasets using an electronic nose, then classified and regressed the established BP model. The prediction results based on the BP model are shown in Figure 16.

The results show that classification prediction accuracy of the BP model is 100%. In the scatter of the predicted value of ammonia concentration, most of the results are in the range of Y = X ± 10, and the actual and predicted values are mostly less than 10 ppm. From the error analysis, Figure 16d shows the probability density of the absolute error in predicting the ammonia gas volume concentration using the BP model, where the abscissa is the absolute error, the ordinate is the probability density, and the density function is

(4) $$f(x)=\frac{1}{\sqrt{2\pi }\sigma }e^{-\frac{{\left(x-\mu \right)}^{2}}{2\sigma^{2}}},-\infty <x<\infty .$$

Figure 16d shows that the absolute error distribution for the prediction results using the BP model with single output is more concentrated. The standard deviation of the normal distribution of absolute error in ammonia concentration prediction of the BP model with single output and multi-output is 10.37 and 14.51, respectively. Compared with the model with multi-output, the model with single-output can concentrate the prediction error in a smaller interval, reduce the overall prediction error of the model, and further improve the prediction accuracy.

Under the same gas concentration, the gas flow on the sensor surface varies, resulting in varied sensor responses. Previous studies [31,32,33,34,35] did not consider the electronic nose gas chamber’s effect on the output of the system. In this system, four groups of sensors with cross-sensitivity provide various response signals to the same gas concentration when activated via a bionic chamber. The sensor’s response data can be processed using algorithms, enabling the gas concentration to be correctly determined.

The electronic nose system proposed, based on a bionic chamber, is believed to enhance the precision of gas detection, as per theoretical research and experimental validation. Future studies should include using additional gas source sensors, such as hydrogen sulfide gas sensors, to detect complex gases in livestock and poultry breeding environments and minimize the impact of cross-sensitivity on ammonia detection.

## 4. Conclusions

We developed an innovative active pumping electronic nose detection system, incorporating a biomimetic chamber, specifically tailored for monitoring ammonia concentrations in livestock breeding environments. The design of the biomimetic chamber and the PCB was informed by an analysis of airflow pressure and gas velocity contours, ensuring optimal sensor performance. The experimental results of this system were promising, showcasing a rapid sensor response time of less than 30 s. The system’s array of eight sensors exhibited distinct response amplitudes to both ammonia and ethanol gases, demonstrating their effectiveness in a controlled environment. Following the preprocessing of the output signals, the study employed three models for data classification (i.e., BP, LSTM, and PSO-BP). Remarkably, all three models achieved a perfect prediction accuracy of 100%, highlighting their efficiency in classifying system responses. For single-output regression prediction, the same models were utilized to predict the response of the system. The BP neural network model stood out, achieving an R^2^ value above 0.99, along with the lowest MAE and RMSE. Moreover, this study extended its analysis to multi-output regression prediction, employing BP, DNN, and LSSVM models to predict responses for both ammonia and ethanol gases. The multi-output regression models yielded larger error values compared to the single-output models. This finding suggests an increase in complexity when dealing with multiple outputs; however, the BP model still showed robust performance. The study utilized air as the background gas and did not include any harmful gaseous substances, such as hydrogen sulfide, in the background gas testing. This will be the subsequent phase in the experimentation. Overall, the active pumping electronic nose detection system, equipped with a biomimetic chamber, significantly enhances the detection of ammonia in livestock breeding environments. The utilization of the BP model for both data classification and single-output regression prediction enables accurate, real-time, and stable monitoring of ammonia concentrations. This approach is well-suited to fulfill the technical and functional requirements for environmental monitoring in such settings.

Accuracy and effectiveness are key aspects in gas detection using electronic noses. To address this these, bionics takes a distinctive approach by enhancing the architecture of the electronic nose chamber and the arrangement of sensors based on the shape of the nasal cavity in biological prototypes. The active pumping electronic nose system, which is based on a bionic chamber, enhances the accuracy and effectiveness of gas detection in comparison to the conventional electronic nose detection method. The experimental substance of this work requires optimization. Utilizing space–time information to detect gas concentration values is beneficial for enhancing the accuracy of gas detection and analyzing gas diffusion [41,42]. Subsequently, it is necessary to arrange many electronic nose systems inside the same area in accordance with the surrounding conditions to ascertain the ammonia levels while considering both temporal and spatial factors. Furthermore, it is imperative to counterbalance the impact of temperature and humidity on the sensor using an intelligent algorithm and calibrate the measurement data of the electronic nose through standardized ion chromatography analysis.

## Figures and Tables

**Table 1:** Comparison of evaluation metrics for single-output estimation models of gas volume concentration.

Evaluation Metric	Dataset	Value		
		BP	LSTM	PSO-BP
R^2^	Ammonia training set	0.9939	0.9845	0.9816
	Ammonia test set	0.9923	0.9916	0.9915
	Ethanol training set	0.9993	0.9947	0.9975
	Ethanol test set	0.9959	0.9922	0.9902
MAE	Ammonia training set	4.4204	7.4724	7.8857
	Ammonia test set	5.3105	5.7164	6.0176
	Ethanol training set	1.2643	3.1779	2.3203
	Ethanol test set	2.5202	3.7487	4.1104
RMSE	Ammonia training set	7.7758	11.5302	12.4395
	Ammonia test set	7.8833	9.2732	9.0892
	Ethanol training set	1.6927	4.7793	3.3379
	Ethanol test set	4.1787	5.5147	5.8519

**Table 2:** Comparison of evaluation metrics for multi-output gas volume concentration estimation models.

Evaluation Metric	Dataset	Value		
		BP	LSTM	PSO-BP
R^2^	Ammonia training set	0.9933	0.96339	0.9689
	Ammonia test set	0.9809	0.93654	0.9725
	Ethanol training set	0.9977	0.99073	0.9938
	Ethanol test set	0.9981	0.97795	0.9938
MAE	Ammonia training set	5.6754	9.4706	9.2357
	Ammonia test set	9.5785	15.2078	8.3824
	Ethanol training set	2.3837	4.7802	3.5657
	Ethanol test set	2.3536	7.943	3.5129
RMSE	Ammonia training set	8.0925	17.9713	16.6752
	Ammonia test set	14.2569	24.5642	13.4656
	Ethanol training set	3.0485	6.0374	5.3372
	Ethanol test set	3.1448	10.3046	4.4004

## Data Availability

All raw experimental data involved in the paper are stored at https://doi.org/10.6084/m9.figshare.24770862.v1, https://doi.org/10.6084/m9.figshare.24770238.v1, https://doi.org/10.6084/m9.figshare.25124207.v1, https://doi.org/10.6084/m9.figshare.25100417.v2, accessed on 1 January 2024.
